# Editorial: Cellular and Phenotypic Plasticity in Cancer

**DOI:** 10.3389/fonc.2015.00171

**Published:** 2015-08-04

**Authors:** Petranel T. Ferrao, Andreas Behren, Robin L. Anderson, Erik W. Thompson

**Affiliations:** ^1^Oncogenic Signalling and Growth Control Program and Cancer Therapeutics Program, Peter MacCallum Cancer Centre, East Melbourne, VIC, Australia; ^2^The Sir Peter MacCallum Department of Oncology, The University of Melbourne, East Melbourne, VIC, Australia; ^3^Cancer Immunobiology, Olivia Newton-John Cancer Research Institute, Heidelberg, VIC, Australia; ^4^School of Cancer Medicine, La Trobe University, Heidelberg, VIC, Australia; ^5^Metastasis Research Laboratory, Peter MacCallum Cancer Centre, East Melbourne, VIC, Australia; ^6^Institute of Health and Biomedical Innovation and School of Biomedical Sciences, Queensland University of Technology, Kelvin Grove, QLD, Australia

**Keywords:** cancer, phenotypic plasticity, signaling pathways, drug resistance, epithelial-mesenchymal transition (EMT), cross-talk, exosomes, immune system

Cellular and phenotypic plasticity is a key feature of development and normal function of cells within most multicellular organisms. The ability to respond to various intrinsic and external cues and stimuli in a regulated fashion allows for appropriate cellular adjustments. This plasticity observed in most cell types is retained in cancer and can lead to opportunistic adaptation allowing therapeutic escape and acquisition of motile and invasive abilities that pose ongoing challenges for effective therapy. The dynamic nature of this plasticity and the apparent requirement for widely divergent phenotypes for different aspects of the metastatic cascade (e.g., initial escape – mesenchymal and distant colonization – epithelial) is especially challenging.

The consequences and functional outcomes of this plasticity are well-studied and widely reported in relation to epithelial–mesenchymal transition (EMT). In normal and cancer cells alike, EMT is regulated through signaling pathways (1), the outcome of which is dictated by the balance and cross-talk between the pathways as reviewed within this Research Topic (2). EMT is classically defined as a dynamic, multistep cellular process that allows non-motile, highly organized and polarized epithelial cells to acquire motile and more fibroblast-like, mesenchymal characteristics. It is accompanied by the loss of some epithelial characteristics including specialized cell–cell junctions and apical-basal polarity, and a complex reorganization of the cellular cytoskeleton (3). Functional consequences of EMT, including enhanced or acquired migratory capacity, can result in the release of tumor cells into circulation (4) and ultimately metastasis of the cancer. Not all cells within a tumor undergo EMT, as different signaling inputs at different tumor sites or a variation in genetic drivers in different clones can lead to different cellular states. However, EMT and EMT-like processes contribute greatly to tumor heterogeneity, the challenges of which are highlighted for colorectal cancer within this Research Topic (5). EMT-like phenotype-switching processes have also been described in non-epithelial cancers, such as in melanoma (6). As reviewed in this Research Topic (7), this can involve similar EMT inducers and EMT-transcription factors (EMT-TFs), but variable patterns in terms of expression. Also, unlike the more distinct differences seen after EMT in development, carcinoma systems often exhibit a partial EMT, sometimes called a metastable or hybrid phenotype, reinforcing the concept of dynamic epithelial–mesenchymal plasticity (EMP) (3, 8, 9).

Besides the already mentioned functional changes, tumor cells can undergo changing antigen patterns in a dynamic process parallel to phenotype switching (10), allowing for escape from recognition by already primed cytotoxic T lymphocytes (CTLs) and resulting in immune evasion. Thus, while adaptive as well, the immune system needs to constantly re-adjust, just one of the many obstacles for immune-based tumor control.

Recently, there has been an expanding body of research linking EMT and the mesenchymal-like phenotypic state of cells to therapy resistance. In the case of epithelial cancers, such as breast and colorectal cancers, elevated expression of mesenchymal markers or alterations to mesenchymal phenotypes has been shown to be associated with increased metastasis (11) and poor response to treatment (12). Cancer cells in a more mesenchymal-like state are more refractory to conventional cytotoxic therapies, to radiation, and to targeted therapies (13, 14). The exact mechanism of survival in these cells is not yet clear. It is possible that phenotypic mediators can confer survival or anti-apoptotic signals, or possibly the altered phenotypic state usually associated with reduced proliferation renders them refractory to cell death following treatment.

The extent of changes within the proteome and signaling networks of cancer cells, particularly in the context of acquired resistance to targeted therapies (driven by genetic changes), is broad and exemplified in this Research Topic by a phospho-proteomics study of melanoma cells resistant to BRAF inhibitor therapy (15). Experience from the clinic of patients relapsing within months of BRAF-inhibitor treatment demonstrates the challenging clinical implications of this complexity (7).

Cancer cells do not exist in isolation. They are in direct contact with stromal cells and an intense crosstalk between “normal” cells and cancer cells is constantly occurring. The tumor microenvironment is regulated by factors produced by both the tumor and the stromal cells (5) and is not limited to close proximity. In recent years it has been demonstrated that primary tumors can establish favorable conditions for future metastasis in distant organs. The so-called pre-metastatic niche can be formed by non-tumorigenic host cells, cytokines, and tumor-derived exosomes, small extracellular vesicles that transfer information from a tumor to other cells as reviewed by Vella (16), thereby influencing other cellular compartments for promotion of tumor growth and metastasis (17).

Based on these data, it is clear that prevention of a phenotypic switch to this refractory state or reversal to an epithelial-like state (MET) that would restore proliferative capacity may provide a means for maintaining or enhancing drug responsiveness, and possibly even reducing heterogeneity. Hence, identifying the molecular drivers that induce or maintain the mesenchymal-like phenotype, provides an opportunity for “drugging” this phenotypic drug-resistant state, through inhibition of the key signaling pathways that regulate the critical EMT-inducers (12, 18, 19). This approach must be tempered with the risk that reversion to a proliferative MET state will increase tumor burden if the therapies delivered are not sufficiently effective. Our understanding of the balance, regulation, and cross-talk between the pathways intrinsically within the cancer cells, as well as extrinsically in relation to the tumor microenvironment, is revealing opportunities for multi-modality therapies. The identification of potential biomarkers of cell state and drug response will also help to guide the choice of therapy and timing for individualized treatment for each patient. The use of novel, multi-cellular organisms for expanding our understanding of these processes (20) is a necessary path to advance research discoveries in this area.

With a better understanding of the processes implicated in cancer progression, and the key regulatory elements, options for improved therapeutic strategies can be designed to specifically predict and exploit the plasticity of cancer cells. As we move from an era of DNA damaging therapy into an era of combination multi-modality treatments, the focus of the therapeutic target shifts from just the tumor type or the genetic alteration, to the interplay between oncogenic drivers, the vasculature, the microenvironment, and, most promisingly, the immune system. Understanding this complex interplay and the adaptive changes induced by therapy, within the tumor cells as well as within interacting compartments, is an undeniably important aspect of current and future research efforts toward effective treatment to control, and hopefully cure, cancer.

## Conflict of Interest Statement

The authors declare that the research was conducted in the absence of any commercial or financial relationships that could be construed as a potential conflict of interest.
